# Mathematical modeling of wear behavior and Abbott Firestone zones of 0.16C steel using response surface methodology

**DOI:** 10.1038/s41598-022-18637-3

**Published:** 2022-08-25

**Authors:** Ramadan N. Elshaer, Mohamed K. El-Fawakhry, Taha Mattar, Ahmed I. Z. Farahat

**Affiliations:** 1 Tabbin Institute for Metallurgical Studies, Cairo, Egypt; 2 Central Metallurgical R&D Institute, Cairo, Egypt

**Keywords:** Engineering, Materials science

## Abstract

The effects of applied pressure and running velocity on wear behavior as well as Abbott Firestone zones of low carbon steel (0.16C) were evaluated using response surface methodology (RSM). At room temperature, three different pressures (0.5, 1.5, and 2.5 MPa) and three different velocities (1.5, 2.25, and 3 m/s) were used to conduct dry sliding wear trials utilizing the pin-on-disc method according to the experimental design technique (EDT). The experiments were created using central composite design (CCD) as a starting point. The relationship between input factors (pressure and velocity) and responses (wear rate and Abbott Firestone zones) of 0.16C steel was demonstrated using analysis of variance (ANOVA). The best models for wear rate as well as Abbott Firestone zones produced accurate data that could be estimated, saving time and cost. The results revealed that pressure had the greatest impact on the alloy’s dry sliding wear behavior of the two variables studied. In general, the predicted result shows close agreement with experimental results and hence created models could be utilized for the prediction of wear behavior and Abbott Firestone zones satisfactorily.

## Introduction

Wear properties of various types of steel have long been employed as a critical mechanical property in tribo-system technical design, a variety of engineering applications, and lifespan prediction^1^. However, defining the wear properties for various types of steel is difficult because they are dependent on a variety of elements such as contact type as well as kinetic and environmental factors. Nonetheless, a number of studies^1–4^ have investigated the effects of running velocity speed, contact force as well as temperature on the wear behavior of diverse industrial materials and their applications in tribo-system construction. This could be owing to the fact that each of these important components has its own set of wear curves, at the very least, accurately reflecting the material’s wear behavior. However, even though this is significant in real tribo-systems. So, these typical wear curves do not reveal the influence of a shift in sliding conditions, like sliding speed or load, on material wear behavior. Typically for wear testing, process variables are applied pressure, running velocity, and time. Under dry sliding conditions, Narayanan et al.^5^ evaluated the effect of process variables (load, velocity, and distance) on the wear behavior of Ti–3Al–2.5V alloy. They found that these factors make significant contributions to wear properties.

Design of experiments (DOE) methods for example factorial design (FD), response surface methodology (RSM), as well as Taguchi methods, are now commonly utilized instead of the time-consuming and expensive one-factor-at-a-time experimental approach. RSM was employed to improve the wear results. This method involves employing modeling approaches to determine the link between input and outcome variables for experiments. To do so effectively, you’ll need a mathematical model that can anticipate the response output based on the influences of many process variables, especially when assessing material properties. DOE, analysis of variance (ANOVA), as well as regression analysis, can all be put used to predict mechanical and tribological features^6^.

Sliding is a powerful tool to investigate the machining process for high-performance devices. Scratching at a nanoscale depth of cut is normally performed at μm/s or mm/s, which is three to six orders of magnitude lower than those used in pragmatic machining processes. A novel method of single grain sliding was conducted on a developed grinder, which was carried out at 40.2 m/s and nanoscale depth of cut^7^ Force, stress, depth of cut, and size of plastic deformation are calculated. This method opens a new pathway to investigate the fundamental mechanism of abrasive machining, such as cutting, grinding and polishing, etc.^8,9^. In addition, a novel model for the maximum undeformed chip thickness is proposed, which is in good agreement with those experimental results^10^. Under the breakthrough of theories, novel machining methods and tools are developed^11^. These studies are a great contribution to the tribological field and manufacturing industry^8^.

RSM is a powerful tool (statistical and mathematical model) that may be used to construct an empirical equation for predicting wear and better understanding wear behavior in terms of pressure, velocity, and time of applied factors. RSM has become a widespread practice in engineering challenges as well as it was extensively employed in the characterization of problems where input factor affects some performance of output factors. RSM gives quantitative measures of potential factor interactions that are difficult to achieve with other optimization techniques. When dealing with multi-variable responses, RSM is the proper approach to use. The number of trials needed to respond to a model is greatly reduced using this strategy. The authors looked into using RSM to improve process characteristics^12,13^.

Kumar et al.^14^ investigated the influence of load, sliding distance, and velocity size range of reinforcement on wear behavior of Al–Si–Mg alloy. They observed that wear rate was found to decrease and then increase with increasing wt.% of reinforcement and wear rate was found to increase with increase in the sliding distance but wear rate was found to decrease with increase in sliding velocity. They developed RSM model to forecast wear rate of Al–Si–Mg alloy reinforced with B_4_C/Al_2_O_3_. Elshaer et al.^15^ and Rajmohan et al.^16^ observed that wear rate increases with increasing load. Rajmohan et al.^16^ employed ANOVA to investigate the wear behavior of composites and discovered that load is a key determinant in composite wear. Soumaya et al.^17^ studied the effect of load (P) and linear sliding speed (V) on wear behavior and friction coefficient of 13Cr5Ni2Mo steel. They developed a mathematical model that allowed them to predict the wear behavior based on test parameters (P and V). Also, Narayanan^5^ investigated the influence of process factors on wear loss using RSM—based mathematical models. They used ANOVA to analyze optimal combination of process parameters that minimize the wear loss is determined. They indicated that among all three factors, the most important aspect influencing the alloy’s dry sliding wear behavior is the normal load. Furthermore, as the usual load and sliding velocity rise, the alloy’s wear rate increases. Elshaer et al.^18^ investigated the surface texture of Carbon Steel Machine Elements using Abbott Firestone curve.

A statistical tool can be used to predict wear rate values using RSM, and it can also be used to predict the best parameter values to achieve a minimum wear rate, within the given range of experimental parameter values. The current study goal is to create models for predicting wear rate and Abbott Firestone zones (high peaks, exploitation, and voids) as a function of key wear variables (pressure and velocity). The influences of input factors (applied pressure and running velocity) on wear rate and Abbott Firestone zones after hot-rolled and QAM_f_ were put to the test in order to evaluate the DOE-based central composite design (CCD) technique. Quadratic RSM-based predictive models of wear rate as well as Abbott Firestone zones were created and then tested using experimental data.

## Experimental works

### Materials and sample preparation

The low carbon steel (0.16C–0.27Si–1.47Mn–0.02Al), for short 0.16C, used in this study was hot-rolled at 1200 °C (after heating for 30 min) followed by air cooling. The heat treatment process was quenched after martensite finish temperature (QAM_f_) was applied to hot-rolled samples^19^. Wear testing was carried out using pin-on-disk tribometer testing machine under dry state at ambient temperature. Three samples were applied on each condition and the mean was taken. Wear samples having a cylindrical shape of 5 mm diameter and 10 mm length were fixed against high-speed steel (disk wear tool) with surface hardness of 64 HRC. Before each test, desk surface was ground and cleaned with different emery papers up to 1000 grit size. Different running velocities of 1.5, 2.25, and 3 m/s were used with an applied constant pressure of 0.5, 1.5, and 2.5 MPa for 15 min. The sample’s weight was measured before and after the wear testing by electronic scale with 0.1 mg accuracy. The test results were evaluated according to the loss in weight. Worn surfaces of wear tested samples were examined using scanning electron microscope (SEM) and micrographs were analyzed using MATLAB software. By using statistical analysis and Excel software, the final Abbott Firestone curve graphic was created.

### Statistical analysis using RSM

The data obtained (wear rate and worn surface micrographs) from the wear tests were evaluated using Design Expert V13, Response Surface Methodology (RSM) is used in Design of Experiments/statistical analysis software. A collection of mathematical as well as statistical methodologies are referred to as RSM for modeling and evaluating problems in which the goal is to optimize a response that is influenced by multiple variables. So, it is an excellent approach for assessing industrial challenges. Four models have been created, one for wear rate and three for Abbott Firestone curve zones (high peaks, voids, and exploitation). In the RSM, the response and input variables are correlated as follows:

1 $$\text{Y }=\text{ f }\left(\text{A},\text{ B}\right),$$

where Y is the desired response, f is the response function, A is the applied pressure and B is the running velocity.

The researchers utilized a polynomial design of experiments of type P^n^, where “n” represents the number of variables (pressure and velocity) and P represents the number of levels (− 1, 0, + 1). As a result, for each condition, the minimum number of trial tests to be completed is 3^2^ = 9. In this study, the experiment included 13 runs with three levels and two variables using the Experimental Central Composite Design (Table 1). Zero value indicates average value, + 1 indicates maximum limit while—− 1 indicates minimum limit of parameters. To create a mathematical model, the second-order polynomial regression (quadratic, modified) equation was used with two parameters and can be calculated using the formula below.

2 $$\text{R }={\text{b}}_{0}+ {\text{b}}_{1}\text{A }+ {\text{b}}_{2}\text{B }+ {\text{b}}_{3}\text{AB }+ {\text{b}}_{4}{\text{A}}^{2} + {\text{b}}_{5}{\text{B}}^{2} + {\text{b}}_{6}{\text{A}}^{2}\text{B }+ {\text{b}}_{7}{\text{AB}}^{2} ,$$

where R is response (estimated), b_0_ is responses average or intercept coefficient, and b_1_, b_2_……b_7_ are response coefficients, A is pressure and B is velocity.

**Table 1:** Experimental central composite design (CCD).

Std	Run	Factor 1	Factor 2
		A: Pressure, MPa	B: Velocity, m/s
1	3	− 1	− 1
2	9	1	− 1
3	11	− 1	1
4	10	1	1
5	6	− 1	0
6	8	1	0
7	7	0	− 1
8	12	0	1
9	2	0	0
10	4	0	0
11	13	0	0
12	1	0	0
13	5	0	0

Variables	Levels		
	− 1	0	1
Pressure, MPa	0.5	1.5	2.5
Velocity, m/s	1.5	2.25	3

## Results and discussion

### Mathematical modeling for wear rate

#### Wear results

**Figure 1:**
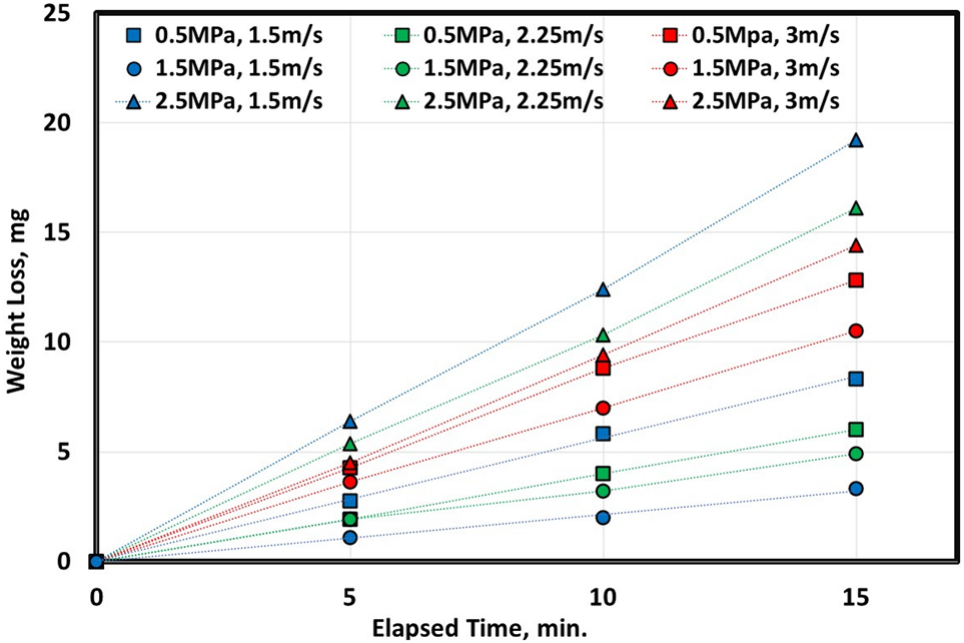
Variation of weight loss with different sliding speeds of 0.16C hot rolled steel.

**Figure 2:**
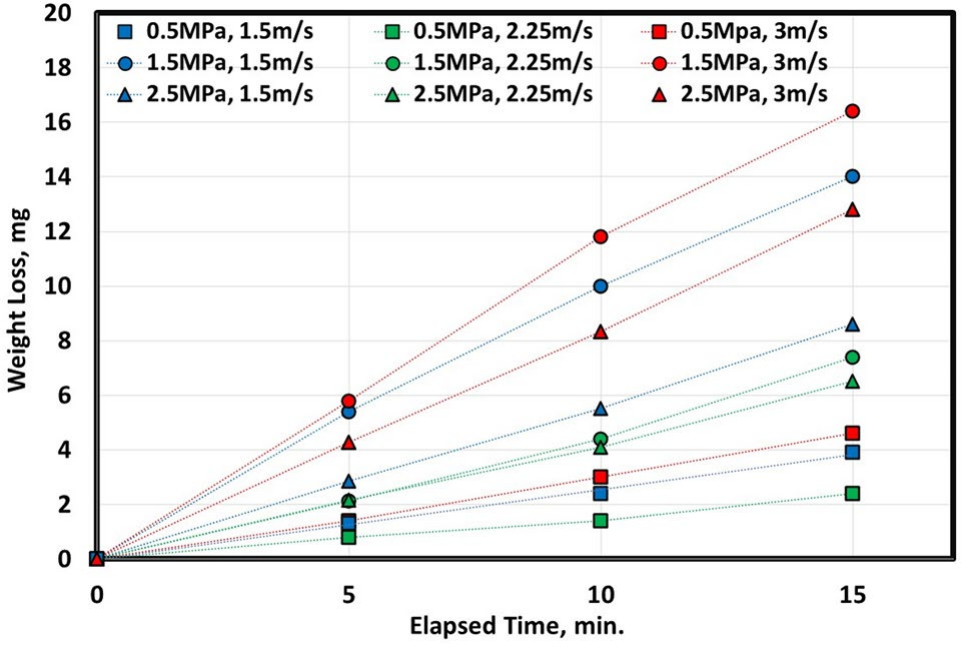
Variation of weight loss with different sliding speeds of 0.16C QAM_f_ steel.

Figures 1 and 2 indicate a relationship between elapsed time (during wear testing) in minutes and weight loss in mg at different pressure in MPa and various velocities in m/sec. It seems clear that increasing elapsed time of experimental wear increases the weight loss of metal. Hot-rolled samples, Fig. 1, have the highest weight loss at both maximum pressure (2.5 MPa) and velocity (3 m/s). However, the lowest weight loss was at both medium pressure (1.5 MPa) and velocity (1.5 m/s). On the other hand, after heat-treated samples, Fig. 2, the highest weight loss at medium pressure (1.5 MPa) and highest velocity (3 m/s). However, the lowest weight loss at the smallest pressure (0.5 MPa) and medium velocity (2.25 m/s).

It is very difficult to differentiate between pressure and/or velocity effect on wear rate using Figs. 1 and 2. Therefore, it is very so essential to study both parameters (pressure and velocity) on wear behavior and to construct a mathematical model expressing wear rate versus pressure and velocity. To demonstrate wear rate behavior due to pressure and velocity, CCD was adopted. Tables 2 and 3 show different limits of two parameters (pressure and velocity), and the corresponding wear rate (response 1).

**Table 2:** Experimental CCD showing wear rate of 0.16C hot rolled steel.

Std	Run	Factor 1	Factor 2	Response 1
		A: Pressure, MPa	B: Velocity, m/s	Wear rate, mg/min
1	3	0.50	1.50	0.559
2	9	2.50	1.50	1.272
3	11	0.50	3.00	0.859
4	10	2.50	3.00	0.897
5	6	0.50	2.25	0.337
6	8	2.50	2.25	1.065
7	7	1.50	1.50	0.216
8	12	1.50	3.00	0.791
9	2	1.50	2.25	0.376
10	4	1.50	2.25	0.376
11	13	1.50	2.25	0.376
12	1	1.50	2.25	0.376
13	5	1.50	2.25	0.376

**Table 3:** Experimental CCD showing wear rate of 0.16C QAM_f_ steel.

Std	Run	Factor 1	Factor 2	Response 1
		A: Pressure, MPa	B: Velocity, m/s	Wear rate, mg/min
1	3	0.50	1.50	0.256
2	9	2.50	1.50	0.569
3	11	0.50	3.00	0.284
4	10	2.50	3.00	0.849
5	6	0.50	2.25	0.156
6	8	2.50	2.25	0.429
7	7	1.50	1.50	1.095
8	12	1.50	3.00	1.073
9	2	1.50	2.25	0.429
10	4	1.50	2.25	0.429
11	13	1.50	2.25	0.429
12	1	1.50	2.25	0.429
13	5	1.50	2.25	0.429

#### Statistical analysis of wear rate

The influence of pressure and velocity on the wear rate of 0.16C steel (hot rolled and QAM_f_) is studied in this section. RSM was utilized to perform the analysis and construct wear rate models of 0.16C steel. After proceeding with several trials using Design-Expert software, quadratic models were proposed based on the statistical evaluation of several models, as shown in Tables 4 and 5. The best model is the modified quadratic one which gives a high adjusted correlation factor. Furthermore, the software found that the cubic model was aliased for data ranges acquired. For the wear rate of hot-rolled and QAM_f_ steels, the modified quadratic model (suggested model) was adapted where R-squared values of 0.8723 and 0.9496, respectively. However, adjusted R-squared values of 0.7810 and 0.9135, respectively.

**Table 4:** Model summary statistics of wear rate for hot rolled steel.

Source	Sequential p-value	Std. dev.	R^2^	Adjusted R^2^	PRESS	Recommendation
Linear	**0.0160**	0.3037	0.3058	0.1670	2.03	
2FI	0.4963	0.2997	0.3916	0.1888	3.99	
Quadratic	**0.0003**	**0.1557**	**0.8723**	**0.7810**	**1.72**	**Suggested**
Cubic		0.0253	0.9976	0.9942	0.3733	Aliased

Significant values are in bold.

**Table 5:** Model summary statistics of wear rate for QAM_f_ steel.

Source	Sequential p-value	Std. dev.	R^2^	Adjusted R^2^	PRESS	Recommendation
Linear	0.0376	1.16	0.4812	0.3774	23.76	
2FI	0.9381	1.22	0.4815	0.3087	26.21	
Quadratic	**0.0003**	**0.4308**	**0.9496**	**0.9135**	**12.91**	**Suggested**
Cubic	0.0015	0.1380	0.9963	0.9911	11.06	Aliased

Significant values are in bold.

#### ANOVA of wear rate

ANOVA is a statistical design tool for separating individual effects of the variables under control. The interpretation of the experimental results is carried out by analysis of average and ANOVA. It’s usually done with experimental data to find statistically significant control factors. The effects of applied pressure (P) and running velocity (T) on the wear rate of 0.16C hot rolled and QAM_f_ steels were statistically analyzed using DOE software with a response surface approach and an empirical wear rate model was created based on these effects. The regression model’s significance was tested using the sequential F-test. Tables 6 and 7 show the ANOVA generated model of wear rate. The model’s importance is confirmed by its F-values of 294.64 for hot rolled and 192.52 for QAM_f_. The “P > F” values for the models (hot rolling and QAM_f_) are less than 0.05, indicating that they are significant. This is desirable since it demonstrates that the model parameters have a considerable impact on the response (wear rate). Significant model terms include A, B, AB, A^2^, B^2^, A^2^B, and AB^2^. The model terms aren’t important if the value exceeds 0.1. The model terms that aren’t important can be deleted, perhaps improving the model.

**Table 6:** ANOVA results of quadratic model (wear rate is response) for hot rolled steel.

Source	Sum of squares	df	Mean square	F-value	p-value	
Model	1.33	7	0.1893	294.64	< 0.0001	Significant
A-Pressure	0.2650	1	0.2650	412.44	< 0.0001	
B-Velocity	0.1653	1	0.1653	257.30	< 0.0001	
AB	0.1139	1	0.1139	177.29	< 0.0001	
A^2^	0.3539	1	0.3539	550.78	< 0.0001	
B^2^	0.0711	1	0.0711	110.67	0.0001	
A^2^B	0.1251	1	0.1251	194.64	< 0.0001	
AB^2^	0.0414	1	0.0414	64.47	0.0005	
Residual	0.0032	5	0.0006			
Lack of fit	0.0032	1	0.0032			
Pure error	0.0000	4	0.0000			
Cor total	1.33	12				
Std. dev.	0.0253		R^2^		0.9976	
Mean	0.6058		Adjusted R^2^		0.9942	
C.V.%	4.18		Predicted R^2^		0.7190	
PRESS	1.72		Adeq precision		51.3323

**Table 7:** ANOVA results of quadratic model (wear rate is response) for QAM_f_ steel.

Source	Sum of squares	df	Mean square	F-value	*p*-value	
Model	25.66	7	3.67	192.52	< 0.0001	Significant
A-Pressure	8.32	1	8.32	436.94	< 0.0001	
B-Velocity	0.0002	1	0.0002	0.0092	0.9273	
AB	0.0095	1	0.0095	0.4966	0.5125	
A^2^	9.56	1	9.56	501.93	< 0.0001	
B^2^	6.96	1	6.96	365.66	< 0.0001	
A^2^B	0.0837	1	0.0837	4.40	0.0901	
AB^2^	1.12	1	1.12	58.83	0.0006	
Residual	0.0952	5	0.0190			
Lack of fit	0.0952	1	0.0952			
Pure error	0.0000	4	0.0000			
Cor total	25.76	12				
Std. dev.	0.1380		R^2^		0.9963	
Mean	2.51		Adjusted R^2^		0.9911	
C.V.%	5.50		Predicted R^2^		0.5705	
PRESS	12.91		Adeq precision		50.7807	

The predicted R^2^ values of 0.719 for hot rolled and 0.5705 for QAM_f_, as shown in Tables 6 and 7, are not as close to the adjusted R^2^ values of 0.9942 for hot rolled and 0.9911 for QAM_f_, indicating a difference of over 0.2. This could be a sign of a significant block effect or a problem with your model and/or data. The Adeq Precision was 51.332 for hot rolled and 50.781 for QAM_f_, indicating that the model can navigate the design space, as a ratio larger than 4 is ideal. The R^2^ values of 0.9976 for hot rolled and 0.9963 for QAM_f_ indicate that the variability of responses was 99.76 and 99.63%, respectively, around the mean, demonstrating that the model fit the data well. The model’s lack of fits is negligible, this demonstrates that the proposed model fits well Among the parameter ranges studied, wear rate, as a result, can be determined by the final empirical Eqs. (3) and (4) in terms of actual factors, pressure (P), velocity (V) their multiplication products.

3 $$\text{Wear rate }\left(\text{hot rolled}\right)= 4.89469 - 4.54609 \times \text{ P }- 3.59650 \times \text{ V }+ 2.41000 \times \text{ P }\times \text{ V }+ 1.27670 \times {\text{P}}^{2} + 0.755241 \times {\text{V}}^{2} - 0.408333 \times {\text{P}}^{2} \times \text{ V }- 0.313333\text{ P }\times {\text{V}}^{2},$$

4 $$\frac{1}{\text{Wear rate}}\left({\text{QAM}}_{\text{f}}\right)= -15.59265 - 1.47991 \times \text{ P }+ 23.05962 \times \text{ V }- 6.39556 \times \text{ P }\times \text{ V }+ 2.61189 \times {\text{P}}^{2} - 5.26701 \times {\text{V}}^{2} - 0.334062 \times {\text{P}}^{2} \times \text{ V }+ 1.62954 \times \text{ P }\times {\text{V}}^{2}.$$

#### Wear rate graphs

**Figure 3:**
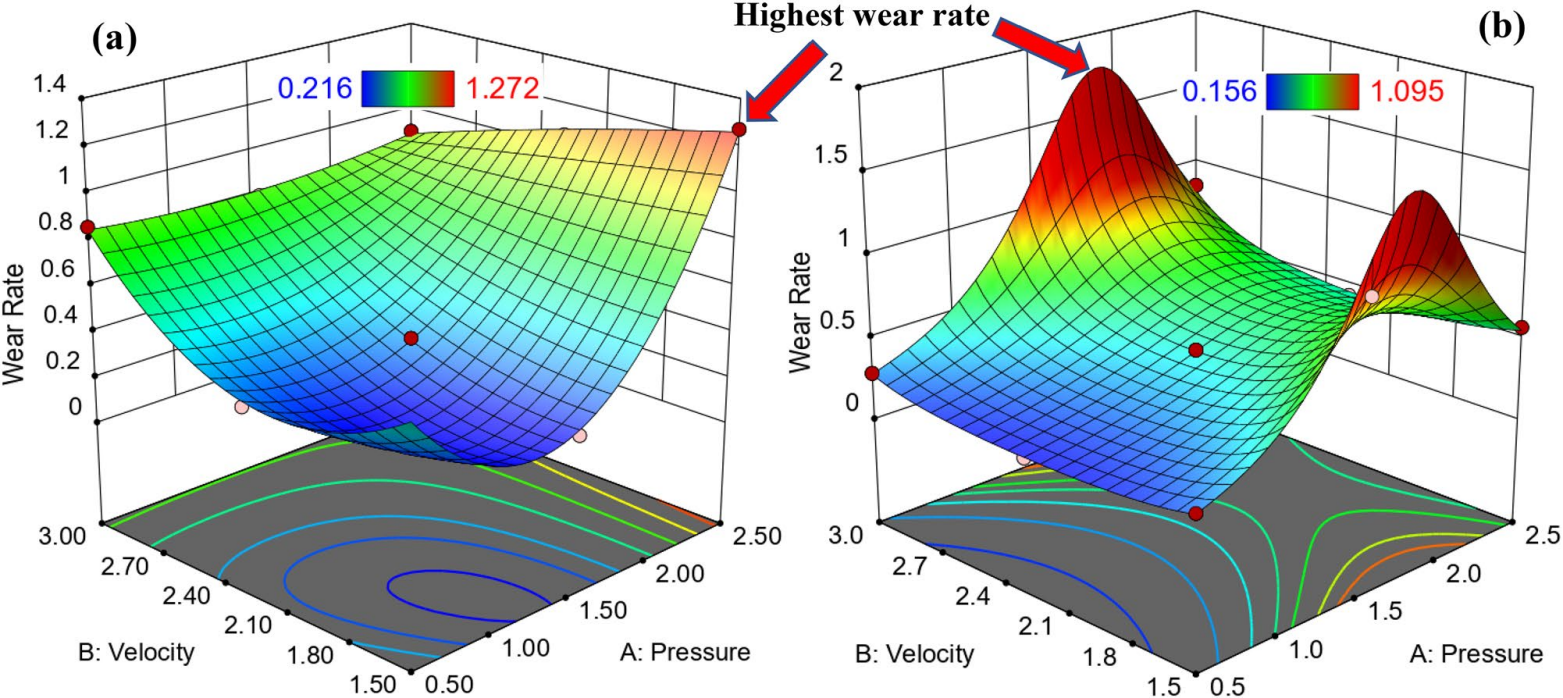
3D surface plot for wear rate relating to pressure and velocity: (**a**) hot-rolled and (**b**) QAM_f_.

**Figure 4:**
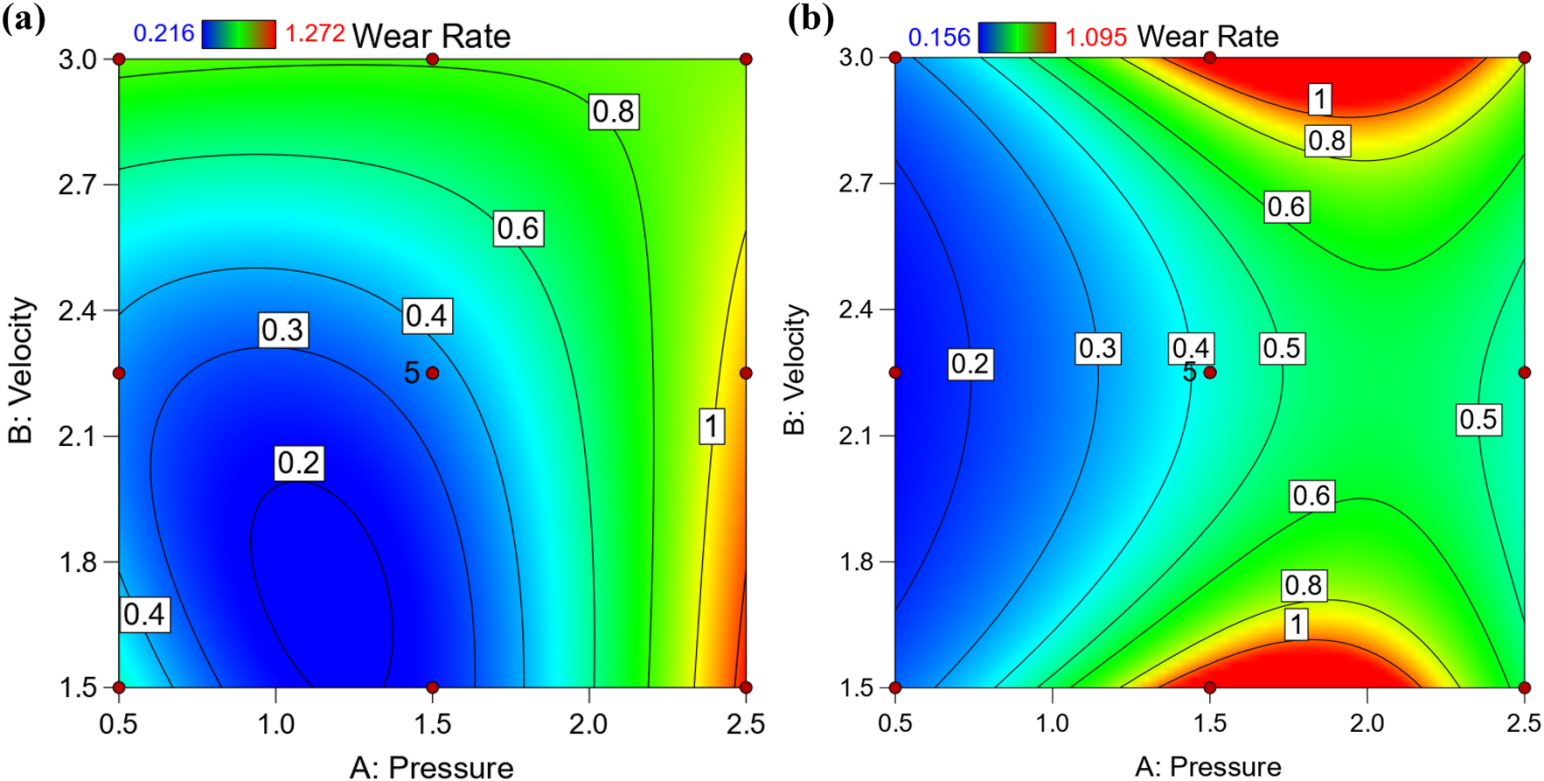
Contour map of wear rate in terms of pressure and velocity: (**a**) hot-rolled and (**b**) QAM_f_.

**Figure 5:**
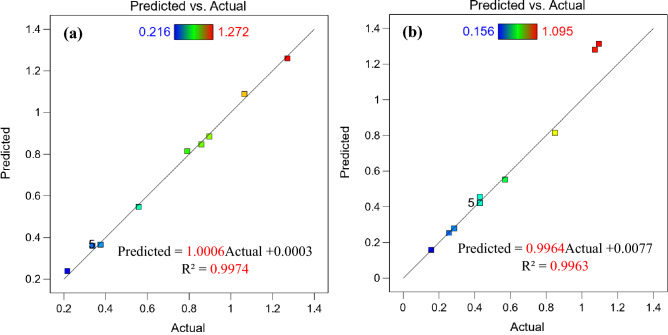
Relationship between actual and predicted wear rate (**a**) hot-rolled and (**b**) QAM_f_.

To follow the exact behavior of wear rate, 3D surface and contour map should be constructed using empirical equation. Figure 3 is 3D surface relationship showing the maximum wear rate at high pressure and low velocity for hot rolled and QAM_f_ samples. On the other hand, wear rate is minimum and constant at high velocity even with changing high pressure. To predict the different values of wear rate it is very useful contour map as seen in Fig. 4. For hot-rolled samples, at pressure of 1.25 MPa, increasing velocity gradually increases wear rate, while at low velocity (beyond 1.25 MPa) increasing pressure gradually increases wear rate. On the other hand, at high pressure (beyond 2 MPa) increasing velocity exhibits a constant wear rate. For, QAM_f_, at low pressure till 1 MPa, increasing velocity gradually increases wear rate, while at constant velocity with increasing pressure gradually until 1.75 MPa increases wear rate. However, at low and high velocities, increasing pressure gradually (until 2.25 MPa) increases the wear rate. Figure 5 shows the relationship between actual and predicted wear rate. This figure indicates empirical equation of predicated weight loss has good fitness with actual weight loss values. It is clear that wear rate decreases due to surface ironing (strain hardening). On the other hand, the wear rate increases due to the ferrite net (soft phase).

### Mathematical modeling of Abbott Firestone zones

#### Abbott Firestone results

**Figure 6:**
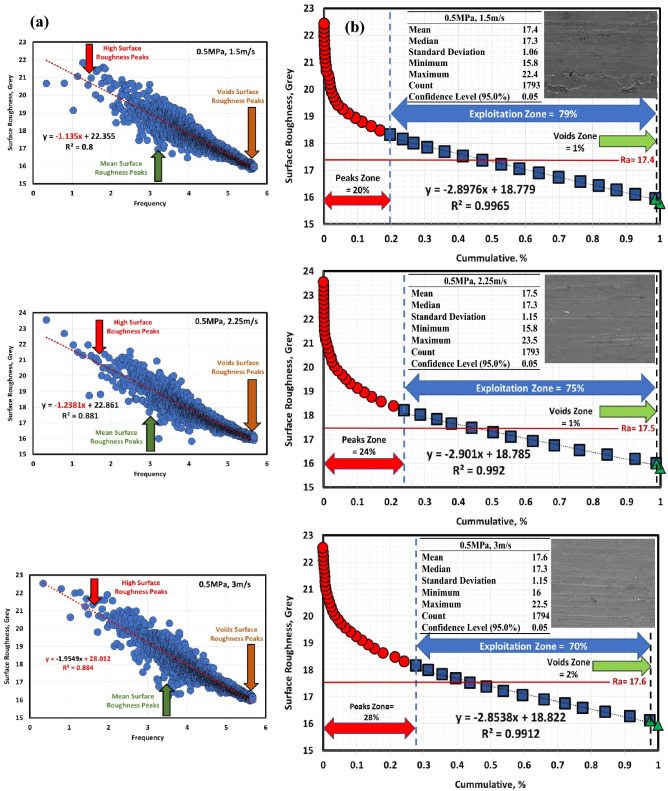
(**a**) Surface texture, (**a,b**) Abbott Firestone curves at 0.5 MPa and different velocities of hot-rolled.

**Figure 7:**
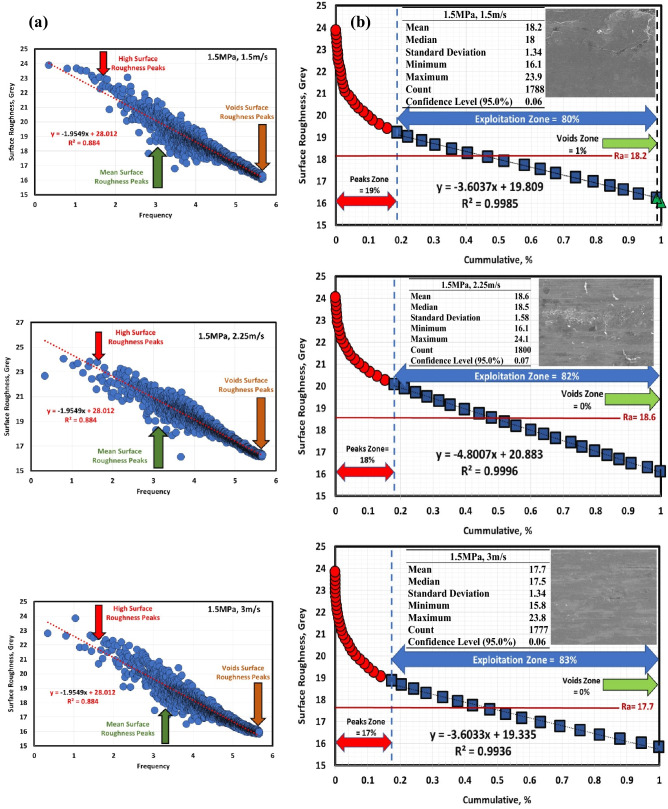
(**a**) Surface texture and& (**b**) Abbott Firestone curves at 1.5 MPa and different velocities of hot-rolled.

**Figure 8:**
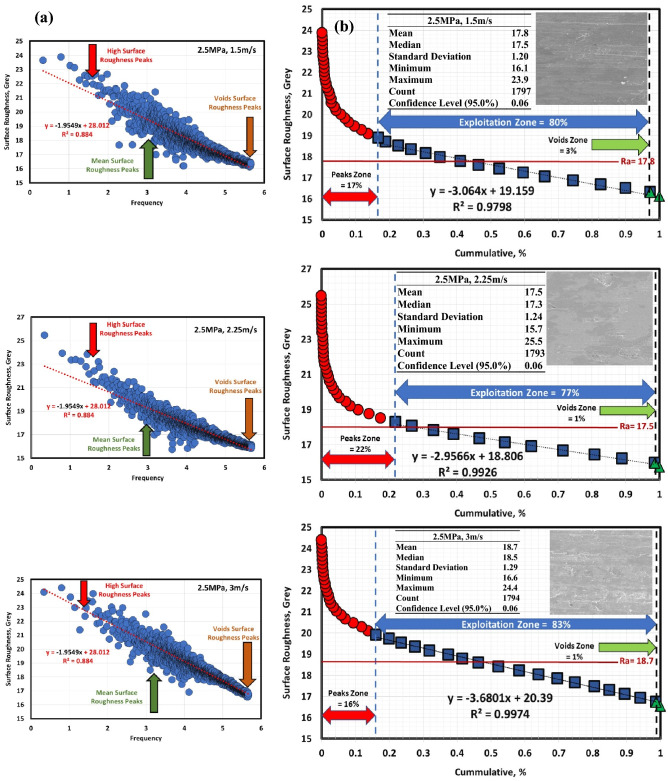
(**a**) Surface texture and (**b**) Abbott Firestone curves at 2.5 MPa and different velocities of hot-rolled.

**Figure 9:**
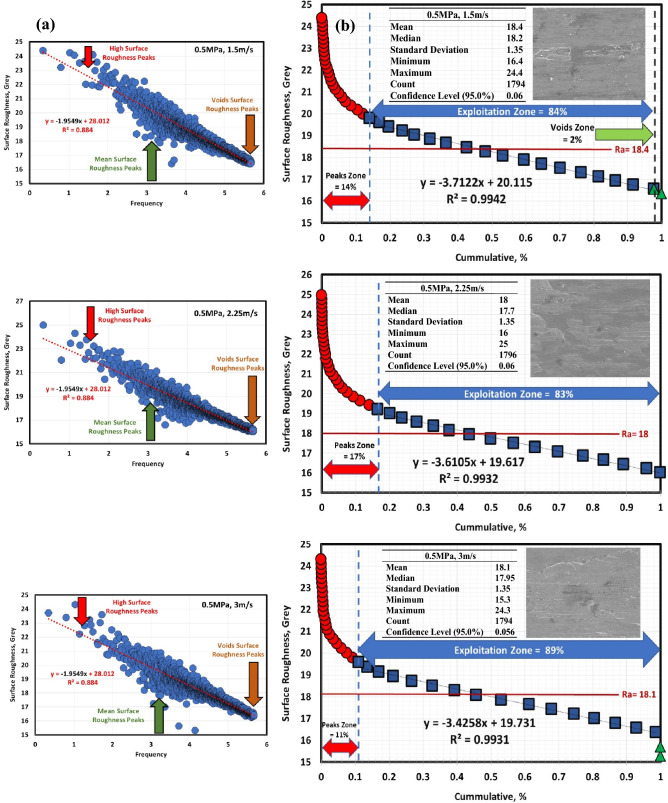
(**a**) Surface texture and (**b**) Abbott Firestone curves at 0.5 MPa and different velocities of QAM_f_.

**Figure 10:**
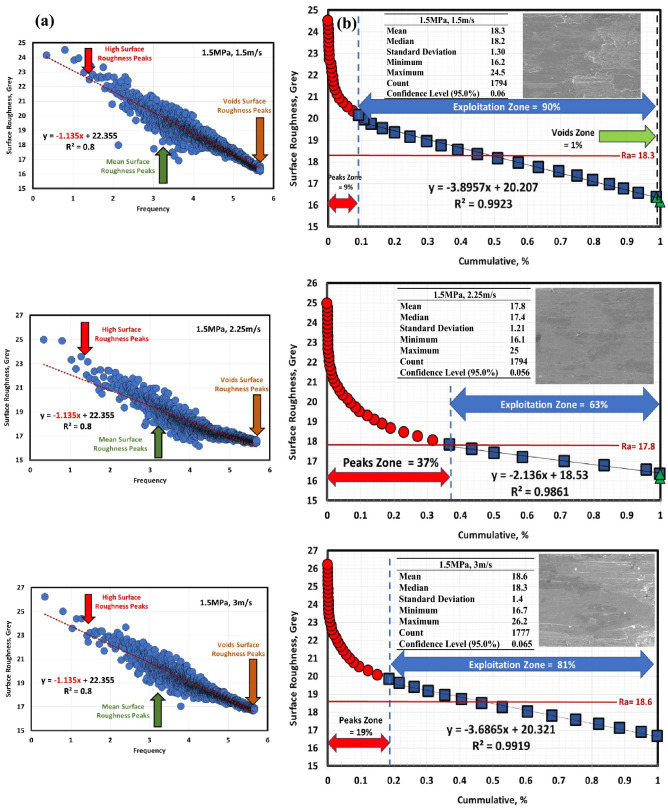
(**a**) Surface texture and (**b**) Abbott Firestone curves at 1.5 MPa and different velocities of QAM_f_.

**Figure 11:**
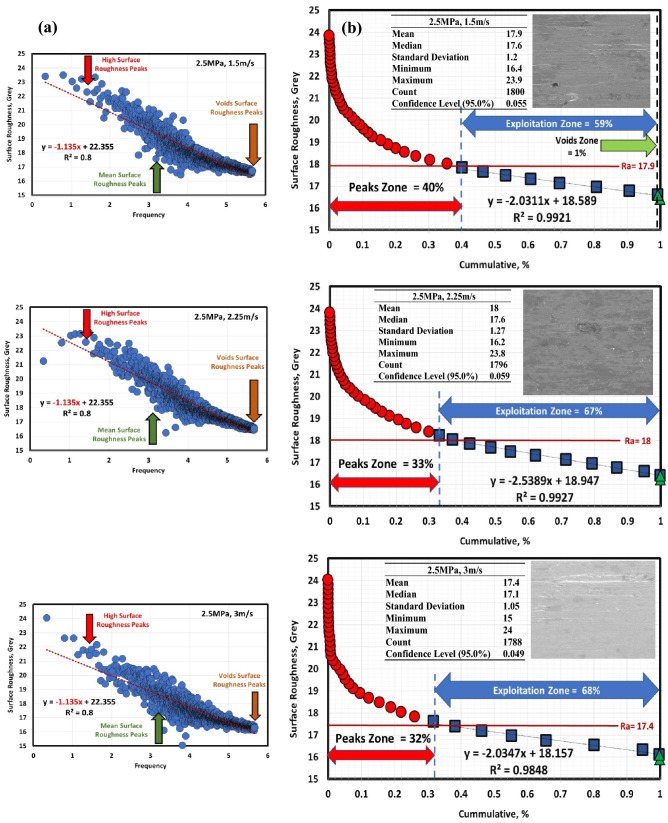
(**a**) Surface texture and (**b**) Abbott Firestone curves at 2.5 MPa and different velocities of QAM_f_.

Figures 6a, 7, 8, 9, 10 and 11a describe the relationship between surface roughness (in grey) and its frequency after hot-rolled and QAM_f_. These figures are qualitatively description. They show three regions of surface roughness such as high, exploitation (mean zone), and low peaks (voids). Therefore, it was necessary to find out the relationship between surface roughness and its distribution quantitively. Figures 6b, 7, 8, 9, 10 and 11b indicate the different three zones high peaks, exploitation zone, and finally voids zone (low peaks) after hot-rolled and QAM_f_.

For hot-rolled, at low pressure, with increasing velocity (1.5–3 m/s), the high peaks zone gradually increases while exploitation zone gradually decreases, see Fig. 6b. This means high peaks increases at the expense of exploitation zone. Furthermore, voids zone is approximately constant. At moderate pressure, with increasing velocity (1.5–3 m/s), high peaks slightly decrease (19–17%), see Fig. 7b. On contrary, the exploitation zone slightly increases (80–83%). It found that voids zone almost zero values. Figure 8b describes the surface roughness of hot rolled steel at high pressure. High peaks show gradual increase followed by gradual decrease (tipping point at 2.25 m/s). Exploitation zone exhibits gradual decrease followed by gradual increase (tipping point at 2.25 m/s). However, the voids zone is 3% at low velocity and decreases to 1% at medium and high velocities.

For QAM_f_, at low pressure, with increasing velocity (1.5–3 m/s), high peaks zone demonstrates gradual increase followed by gradual decrease (tipping point at 2.25 m/s). On the other hand, exploitation zone is approximately constant (84 and 83%) at 1.5 and 2.25 m/s but it increases to 89% at 3 m/s, see Fig. 9b. This means that exploitation zone suffers as high peaks drop. However, voids zone is 2% at low velocity (1.5 m/s) and zero at medium and high velocity (2.25 and 3 m/s). At moderate pressure, with increasing velocity (1.5–3 m/s), high peaks show significant increase followed by significant decrease (tipping point at 2.25 m/s), see Fig. 10b Exploitation zone exhibits gradual decrease followed by gradual increase (tipping point at 2.25 m/s). It was found that voids zone had almost zero value. Surface roughness of QAMf steel at high pressure is seen in Fig. 11b. Zone of high peaks exhibits progressive decrease (40–32%), whereas exploitation zone shows progressive increase (59–68%). It was also discovered that voids zone had nearly zero values. Tables 8 and 9 demonstrate different limits of two parameters (pressure and velocity) and Abbott Firestone zones, with high peaks zone being response 2, exploitation zone being response 3, and voids zone being response 4.

**Table 8:** Experimental CCD showing Abbott Firestone zones of 0.16C hot-rolled steel.

Std	Run	Factor 1	Factor 2	Response 2	Response 3	Response 4
		A: Pressure, MPa	B: Velocity, m/s	High peaks,%	Exploitation zone,%	Voids zone,%
1	3	0.50	1.50	20	79	1
2	9	2.50	1.50	17	80	3
3	11	0.50	3.00	28	70	2
4	10	2.50	3.00	16	83	1
5	6	0.50	2.25	24	75	1
6	8	2.50	2.25	22	77	1
7	7	1.50	1.50	19	80	1
8	12	1.50	3.00	17	83	0
9	2	1.50	2.25	18	82	0
10	4	1.50	2.25	18	82	0
11	13	1.50	2.25	18	82	0
12	1	1.50	2.25	18	82	0
13	5	1.50	2.25	18	82	0

**Table 9:** Experimental CCD showing Abbott Firestone zones of 0.16C QAM_f_ steel.

Std	Run	Factor 1	Factor 2	Response 2	Response 3	Response 4
		A: Pressure, MPa	B: Velocity, m/s	High peaks,%	Exploitation zone,%	Voids zone,%
1	3	0.50	1.50	14	84	2
2	9	2.50	1.50	40	59	1
3	11	0.50	3.00	11	89	0
4	10	2.50	3.00	32	68	0
5	6	0.50	2.25	17	83	0
6	8	2.50	2.25	33	67	0
7	7	1.50	1.50	9	90	1
8	12	1.50	3.00	19	81	0
9	2	1.50	2.25	37	63	0
10	4	1.50	2.25	37	63	0
11	13	1.50	2.25	37	63	0
12	1	1.50	2.25	37	63	0
13	5	1.50	2.25	37	63	0

#### Statistical analysis of Abbott Firestone zones

The influence of pressure and velocity on worn surface i.e., Abbott Firestone zones of 0.16C steel (hot-rolled and QAM_f_) is studied in this section. Tables 10, 11, 12, 13, 14 and 15 show that quadratic models were suggested based on the statistical evaluation of various models after multiple trials with Design-Expert software. The best model is the modified quadratic, which produces a high adjusted correlation factor. Additionally, the cubic order model was discovered to be aliased for data ranges supplied by software. For Abbott Firestone zones (high peaks, exploitation, and voids) of hot-rolled and QAM_f_ steels, the modified quadratic model (suggested model) was adopted. For hot-rolled, R-squared values of high peaks, exploitation, voids were 0.7838, 0.8594, and 0.97,96, respectively. However, adjusted R-squared values were 0.6293, 0.7590, and 0.9651. On the other hand, for QAM_f_, R-squared values were 0.6658, 0.7136, and 0.9560. However, adjusted R-squared values were 0.4270, 0.5090, and 0.9246.

**Table 10:** Model summary statistics of high peaks zone for hot-rolled steel.

Source	Sequential p-value	Std. dev.	R^2^	Adjusted R^2^	PRESS	Recommendation
Linear	0.0891	0.0148	0.3835	0.2602	0.0044	
2FI	0.1637	0.0139	0.5090	0.3453	0.0043	
Quadratic	**0.0567**	**0.0104**	**0.7838**	**0.6293**	**0.0071**	**Suggested**
Cubic	0.0385	0.0064	0.9412	0.8589	0.0241	Aliased

Significant values are in bold.

**Table 11:** Model summary statistics of exploitation zone for hot-rolled steel.

Source	Sequential p-value	Std. dev.	R^2^	Adjusted R^2^	PRESS	Recommendation
Linear	0.2124	0.0026	0.2664	0.1197	0.0001	
2FI	0.0850	0.0023	0.4820	0.3093	0.0002	
Quadratic	**0.0104**	**0.0014**	**0.8594**	**0.7590**	**0.0001**	**Suggested**
Cubic	0.0078	0.0006	0.9798	0.9516	0.0002	Aliased

Significant values are in bold.

**Table 12:** Model summary statistics of voids zone for hot-rolled steel.

Source	Sequential p-value	Std. dev.	R^2^	Adjusted R^2^	PRESS	Recommendation
Linear	0.6561	0.9734	0.0808	− 0.1030	21.80	
2FI	0.1284	0.8959	0.2991	0.0655	37.92	
Quadratic	** < 0.0001**	**0.1731**	**0.9796**	**0.9651**	**1.99**	**Suggested**
Cubic	0.0191	0.0928	0.9958	0.9900	5.01	Aliased

Significant values are in bold.

**Table 13:** Model summary statistics of high peaks zone for QAM_f_ steel.

Source	Sequential p-value	Std. dev.	R^2^	Adjusted R^2^	PRESS	Recommendation
Linear	0.1390	0.0257	0.3260	0.1913	0.0115	
2FI	0.8120	0.0270	0.3305	0.1073	0.0165	
Quadratic	**0.0879**	**0.0216**	**0.6658**	**0.4270**	**0.0287**	**Suggested**
Cubic	0.1153	0.0166	0.8592	0.6620	0.1606	Aliased

Significant values are in bold.

**Table 14:** Model summary statistics of exploitation zone for QAM_f_ steel.

Source	Sequential p-value	Std. dev.	R^2^	Adjusted R^2^	PRESS	Recommendation
Linear	**0.0717**	**9.64**	**0.4097**	**0.2916**	**1474.74**	**Suggested**
2FI	0.8480	10.14	0.4122	0.2163	1867.78	
Quadratic	**0.0808**	**8.03**	**0.7136**	**0.5090**	**3414.80**	**Suggested**
Cubic	0.5279	8.36	0.7782	0.4676	40,570.31	Aliased

Significant values are in bold.

**Table 15:** Model summary statistics of voids zone for QAM_f_ steel.

Source	Sequential p-value	Std. dev.	R^2^	Adjusted R^2^	PRESS	Recommendation
Linear	0.0110	0.4400	0.5941	0.5129	4.34	
2FI	0.2777	0.4328	0.6465	0.5287	8.47	
Quadratic	**0.0007**	**0.1731**	**0.9560**	**0.9246**	**1.99**	**Suggested**
Cubic	0.0191	0.0928	0.9910	0.9783	5.01	Aliased

Significant values are in bold.

#### ANOVA of Abbott Firestone zones

Tables 16, 17, 18, 19, 20 and 21 show the models of ANOVA generated for high peaks, exploitation, and voids zones. The model’s importance is confirmed by its F-values. In case of hot-rolled, F-values of 17.81, 27.84, and 170.10 for high peaks, exploitation, and voids, respectively. However, after QAM_f_, F-values were 4.36, 2.51, and 78.32. For hot-rolled, predicted R^2^ values of 0.9614, 0.9750, and 0.9958 for high peaks, exploitation, and voids zones, respectively. However, after QAM_f_, predicted R^2^ values were 0.8592, 0.7782, and 0.9910, which are as close to adjusted R^2^ values of 0.9074, 0.9400, and 0.9900 for hot-rolled and 0.6620, 0.4676, and 0.9783 for QAM_f_. This could indicate a large block effect or a problem with your data or model. In case of hot-rolled, adeq precision values of 15.505, 19.624, and 41.070 for high peaks, exploitation, and voids zones, respectively. On the other hand, after QAM_f_, adeq precision values were 6.423, 5.132, and 27.459, a ratio of more than 4 is optimal for signaling that the model can explore the design space. Final empirical Eqs. (5)–(10) in terms of actual factors, pressure (P), velocity (V), and their multiplication products can determine wear rate among the parameter ranges examined.

**Table 16:** ANOVA results for quadratic model (high peaks zone is response) of hot-rolled steel.

Source	Sum of squares	df	Mean square	F-value	p-value	
Model	130.02	7	18.57	17.81	0.0030	Significant
A-Pressure	2.00	1	2.00	1.92	0.2248	
B-Velocity	2.00	1	2.00	1.92	0.2248	
AB	20.25	1	20.25	19.41	0.0070	
A^2^	37.25	1	37.25	35.71	0.0019	
B^2^	4.87	1	4.87	4.67	0.0832	
A^2^B	10.08	1	10.08	9.67	0.0266	
AB^2^	10.08	1	10.08	9.67	0.0266	
Residual	5.22	5	1.04			
Lack of fit	5.22	1	5.22			
Pure error	0.0000	4	0.0000			
Cor total	135.23	12				
Std. dev.	1.02		R^2^		0.9614	
Mean	19.46		Adjusted R^2^		0.9074	
C.V.%	5.25		Adeq precision		15.5049

**Table 17:** ANOVA results for quadratic model (exploitation zone is response) of hot-rolled steel.

Source	Sum of squares	df	Mean square	F-value	p-value	
Model	168.00	7	24.00	27.84	0.0010	Significant
A-Pressure	2.00	1	2.00	2.32	0.1882	
B-Velocity	4.50	1	4.50	5.22	0.0711	
AB	36.00	1	36.00	41.76	0.0013	
A^2^	63.45	1	63.45	73.60	0.0004	
B^2^	1.38	1	1.38	1.60	0.2615	
A^2^B	12.00	1	12.00	13.92	0.0136	
AB^2^	8.33	1	8.33	9.67	0.0266	
Residual	4.31	5	0.8621			
Lack of fit	4.31	1	4.31			
Pure error	0.0000	4	0.0000			
Cor total	172.31	12				
Std. dev.	0.9285		R^2^		0.9750	
Mean	79.77		Adjusted R^2^		0.9400	
C.V.%	1.16		Adeq precision		19.6238

**Table 18:** ANOVA results for quadratic model (voids zone is response) of hot-rolled steel.

Source	Sum of squares	df	Mean square	F-value	p-value	
Model	10.26	7	1.47	170.10	< 0.0001	Significant
A-Pressure	0.0000	1	0.0000	0.0000	1.0000	
B-Velocity	0.5000	1	0.5000	58.00	0.0006	
AB	2.25	1	2.25	261.00	< 0.0001	
A^2^	3.47	1	3.47	402.38	< 0.0001	
B^2^	1.06	1	1.06	123.43	0.0001	
A^2^B	0.0833	1	0.0833	9.67	0.0266	
AB^2^	0.0833	1	0.0833	9.67	0.0266	
Residual	0.0431	5	0.0086			
Lack of fit	0.0431	1	0.0431			
Pure error	0.0000	4	0.0000			
Cor total	10.31	12				
Std. dev.	0.0928		R^2^		0.9958	
Mean	0.7692		Adjusted R^2^		0.9900	
C.V.%	12.07		Adeq precision		41.0702

**Table 19:** ANOVA results for quadratic model (high peaks zone is response) of QAM_f_.

Source	Sum of squares	df	Mean square	F-value	p-value	
Model	0.0084	7	0.0012	4.36	0.0622	Not significant
A-Pressure	0.0004	1	0.0004	1.47	0.2793	
B-Velocity	0.0017	1	0.0017	6.19	0.0553	
AB	0.0000	1	0.0000	0.1584	0.7071	
A^2^	0.0000	1	0.0000	0.1657	0.7008	
B^2^	0.0031	1	0.0031	11.04	0.0209	
A^2^B	0.0017	1	0.0017	6.14	0.0560	
AB^2^	0.0002	1	0.0002	0.7254	0.4333	
Residual	0.0014	5	0.0003			
Lack of fit	0.0014	1	0.0014			
Pure error	0.0000	4	0.0000			
Cor total	0.0098	12				
Std. dev.	0.0166		R^2^		0.8592	
Mean	0.0467		Adjusted R^2^		0.6620	
C.V.%	35.63		Adeq precision		6.4228

**Table 20:** ANOVA results for quadratic model (exploitation zone is response) of QAM_f_.

Source	Sum of squares	df	Mean square	F-value	p-value	
Model	1224.86	7	174.98	2.51	0.1645	Not significant
A-Pressure	128.00	1	128.00	1.83	0.2337	
B-Velocity	40.50	1	40.50	0.5800	0.4807	
AB	4.00	1	4.00	0.0573	0.8203	
A^2^	3.58	1	3.58	0.0512	0.8299	
B^2^	374.08	1	374.08	5.36	0.0685	
A^2^B	85.33	1	85.33	1.22	0.3193	
AB^2^	16.33	1	16.33	0.2339	0.6491	
Residual	349.14	5	69.83			
Lack of fit	349.14	1	349.14			
Pure error	0.0000	4	0.0000			
Cor total	1574.00	12				
Std. dev.	8.36		R^2^		0.7782	
Mean	72.00		Adjusted R^2^		0.4676	
C.V.%	11.61		Adeq precision		5.1315

**Table 21:** ANOVA results for quadratic model (voids zone is response) of QAM_f_.

Source	Sum of squares	df	Mean square	F-value	p-value	
Model	4.73	7	0.6752	78.32	< 0.0001	Significant
A-Pressure	0.0000	1	0.0000	0.0000	1.0000	
B-Velocity	0.5000	1	0.5000	58.00	0.0006	
AB	0.2500	1	0.2500	29.00	0.0030	
A^2^	0.0402	1	0.0402	4.67	0.0832	
B^2^	1.06	1	1.06	123.43	0.0001	
A^2^B	0.0833	1	0.0833	9.67	0.0266	
AB^2^	0.0833	1	0.0833	9.67	0.0266	
Residual	0.0431	5	0.0086			
Lack of fit	0.0431	1	0.0431			
Pure error	0.0000	4	0.0000			
Cor total	4.77	12				
Std. dev.	0.0928		R^2^		0.9910	
Mean	0.3077		Adjusted R^2^		0.9783	
C.V.%	30.18		Adeq precision		27.4591	

5 $$\text{High peaks }\left(\text{hot rolled}\right)= 27.63147 - 5.26724 \times \text{ P }- 10.96264 \times \text{ V }+ 8.00000 \times \text{ P }\times \text{ V }- 4.57759 \times {\text{P}}^{2} + 4.9732 \times {\text{V}}^{2} + 3.66667 \times {\text{P}}^{2} \times \text{ V }- 4.88889 \times \text{ P }\times {\text{V}}^{2},$$

6 $$\text{Exploitation zone }\left(\text{hot rolled}\right)= 71.23276 + 1.87931 \times \text{ P }+ 11.34483 \times \text{ V }- 4.00000 \times \text{ P }\times \text{V }+ 4.20690 \times {\text{P}}^{2}-5.40996 \times {\text{V}}^{2}- 4.00000 \times {\text{P}}^{2} \times \text{ V }+ 4.44444 \times \text{ P }\times {\text{V}}^{2},$$

7 $$\text{Voids zone }\left(\text{hot rolled}\right)= 1.13578 + 3.38793 \times \text{ P }- 0.382184 \times \text{ V}-4.00000 \times \text{ P }\times \text{ V }+ 0.370690 \times {\text{P}}^{2}+ 0.436782 \times {\text{V}}^{2} + 0.333333 \times {\text{P}}^{2} \times \text{ V }+ 0.444444 \times \text{ P }\times {\text{V}}^{2},$$

8 $$\text{High peaks }\left({\text{QAM}}_{\text{f}}\right)= 0.342129 + 0.218576 \times \text{ P }- 0.338380 \times \text{ V }-0.049006 \times \text{ P }\times \text{ V }-0.111090 \times {\text{P}}^{2} + 0.091783 \times {\text{V}}^{2} + 0.047563 \times {\text{P}}^{2} \times \text{ V }-0.021799\times \text{ P }\times {\text{V}}^{2},$$

9 $$\text{Exploitation zone }\left({\text{QAM}}_{\text{f}}\right)= 196.65517 + 26.08621 \times \text{ P }- 119.10345 \times \text{ V }-2.66667 \times \text{ P }\times \text{V}+22.86207 \times {\text{P}}^{2} + 30.02299 \times {\text{V}}^{2} +10.66667 \times {\text{P}}^{2} \times \text{ V }- 6.22222 \times \text{ P }\times {\text{V}}^{2},$$

10 $$\text{Voids zone }\left({\text{QAM}}_{\text{f}}\right)= 13.51078 - 5.61207 \times \text{ P }- 9.88218 \times \text{ V }+ 3.33333 \times \text{ P }\times \text{ V }+ 0.870690 \times {\text{P}}^{2} +1.77011 \times {\text{V}}^{2} - 0.333333 \times {\text{P}}^{2} \times \text{ V }- 0.444444 \times \text{ P }\times {\text{V}}^{2}.$$

#### Graphical results of Abbott Firestone zones

**Figure 12:**
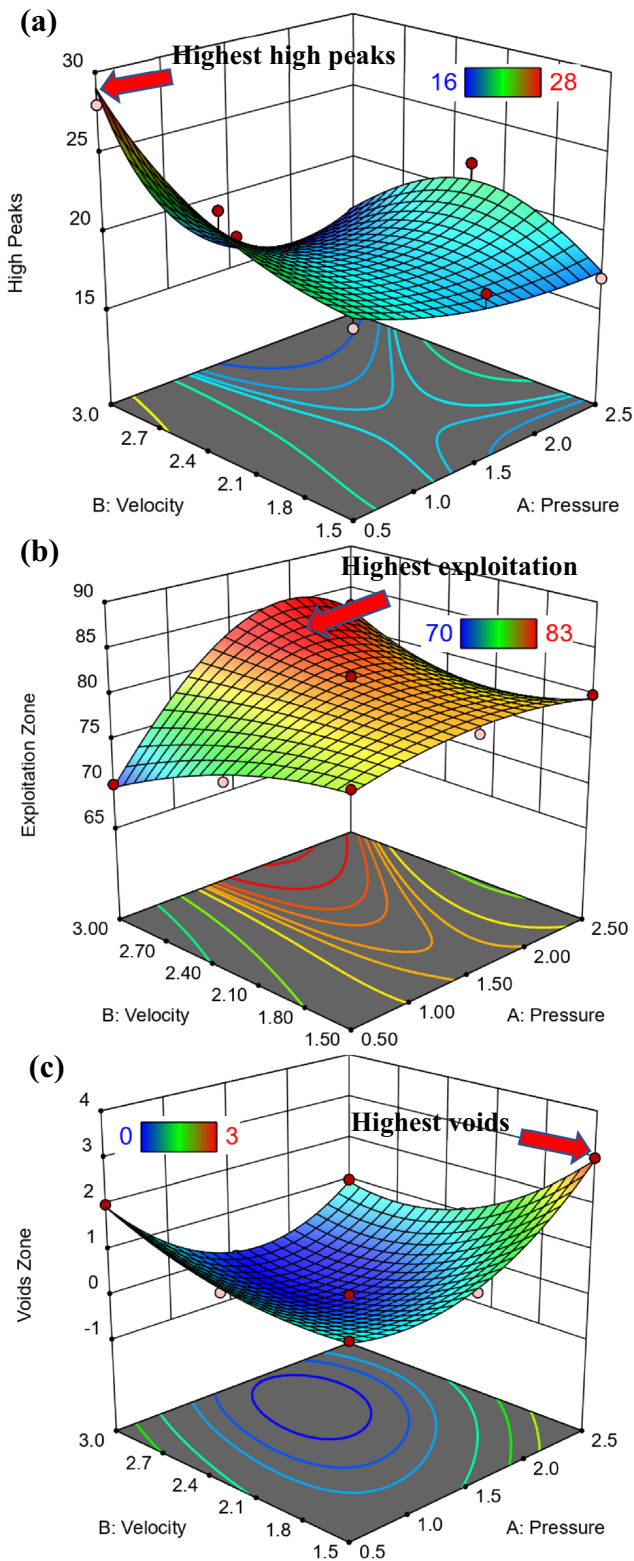
3D Surface plot of (**a**) high peaks, (**b**) exploitation, and (**c**) voids zones of hot-rolled.

**Figure 13:**
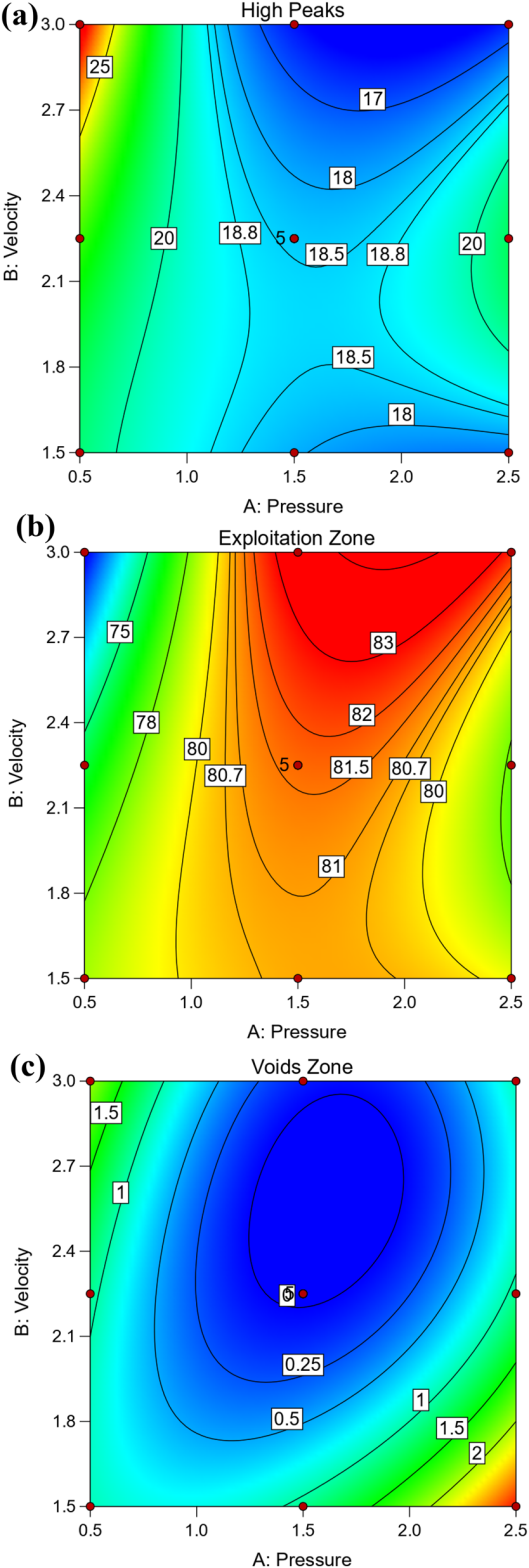
Contour plot of (**a**) high peaks, (**b**) exploitation and (**c**) voids zones of hot-rolled.

Figure 12 shows 3D surface plot of Abbott Firestone zones (high peaks, exploitation, and voids) of hot-rolled steel. The added benefit of 3D graphic is that it allows you to see how the effect of one parameter varies when the value of another change. For instance, considering the effect of velocity (V) at two different values of pressure (P), which were at 0.5 and 2.5 MPa, it is clear that P effect was stronger in first case in high peaks zone. On the contrary for voids zone, it’s worth noting that P effect was stronger in second case. However, the effect of V at 1.5 MPa can be observed that have a stronger effect on exploitation zone, see Fig. 12 (hot-rolled). To predict the different values of Abbott Firestone zones it is very useful contour map as seen in Fig. 13. At low pressure, increasing velocity gradually increases high peaks, while at medium pressure, increasing velocity gradually increases exploitation zone. However, at low velocity, increasing pressure exhibits increases voids zone.

**Figure 14:**
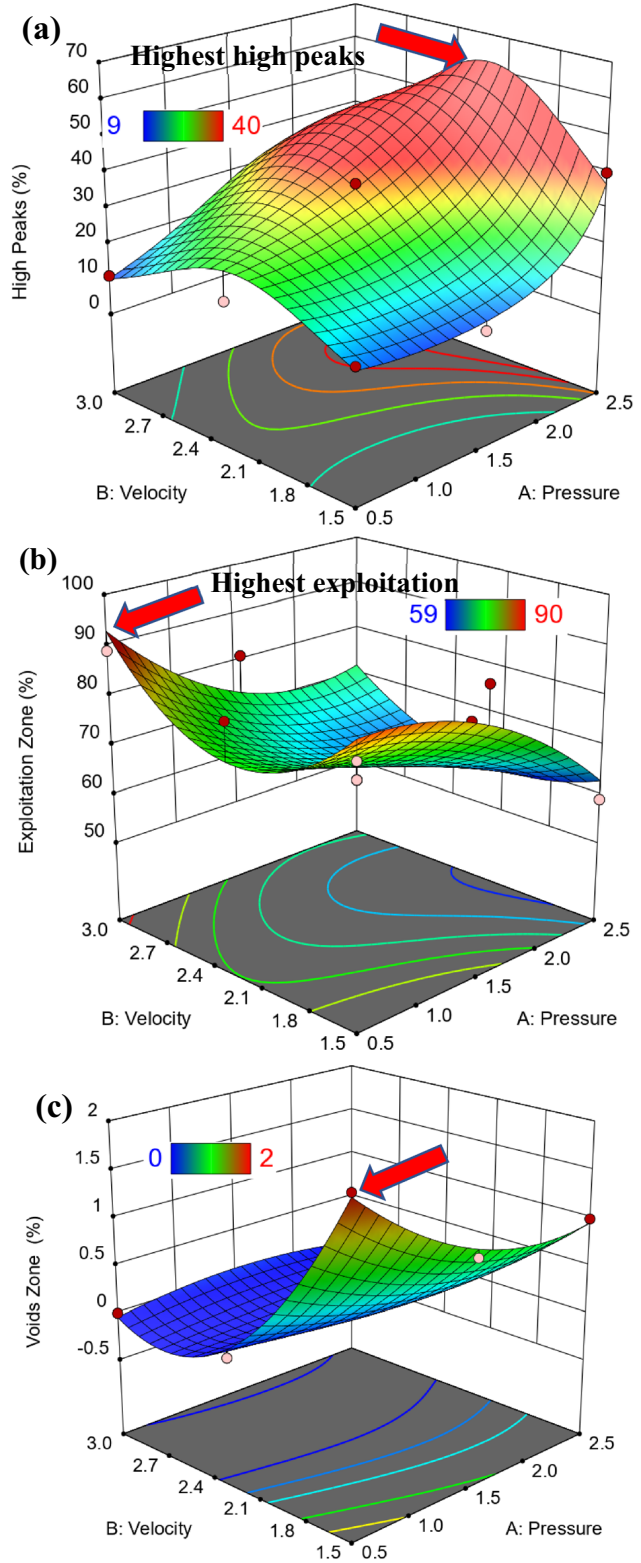
3D Surface plot of (**a**) high peaks, (**b**) exploitation, and (**c**) voids of QAM_f_.

**Figure 15:**
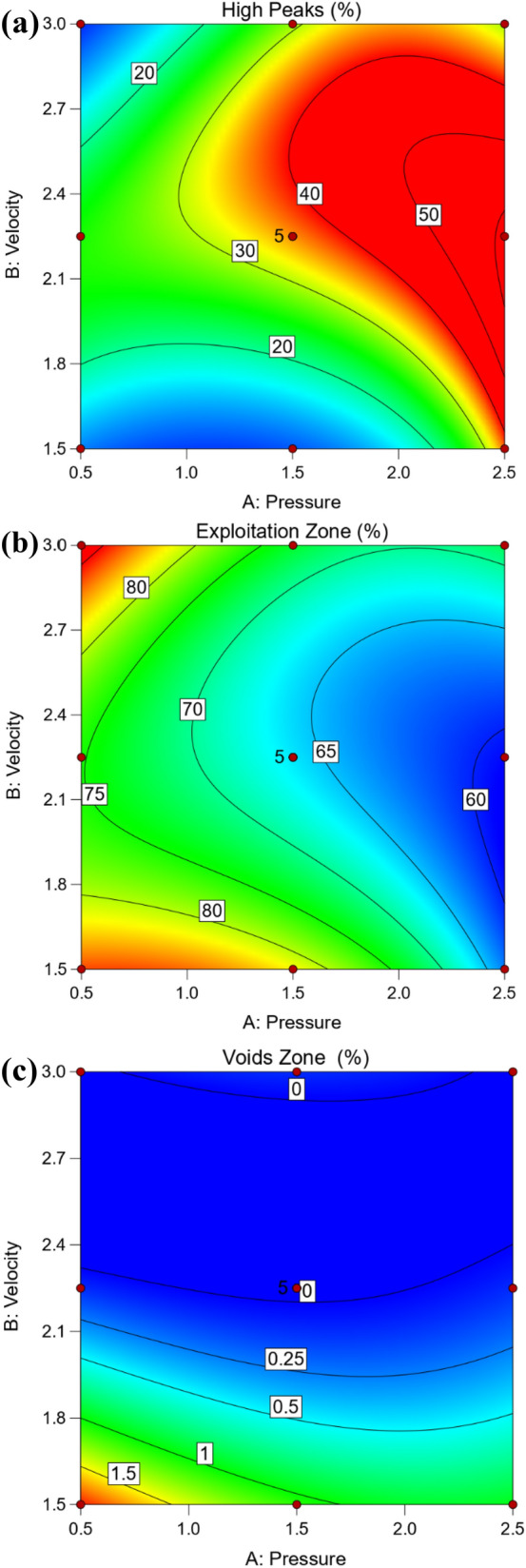
Contour plot of (**a**) high peaks, (**b**) exploitation, and (**c**) voids of QAM_f_.

**Figure 16:**
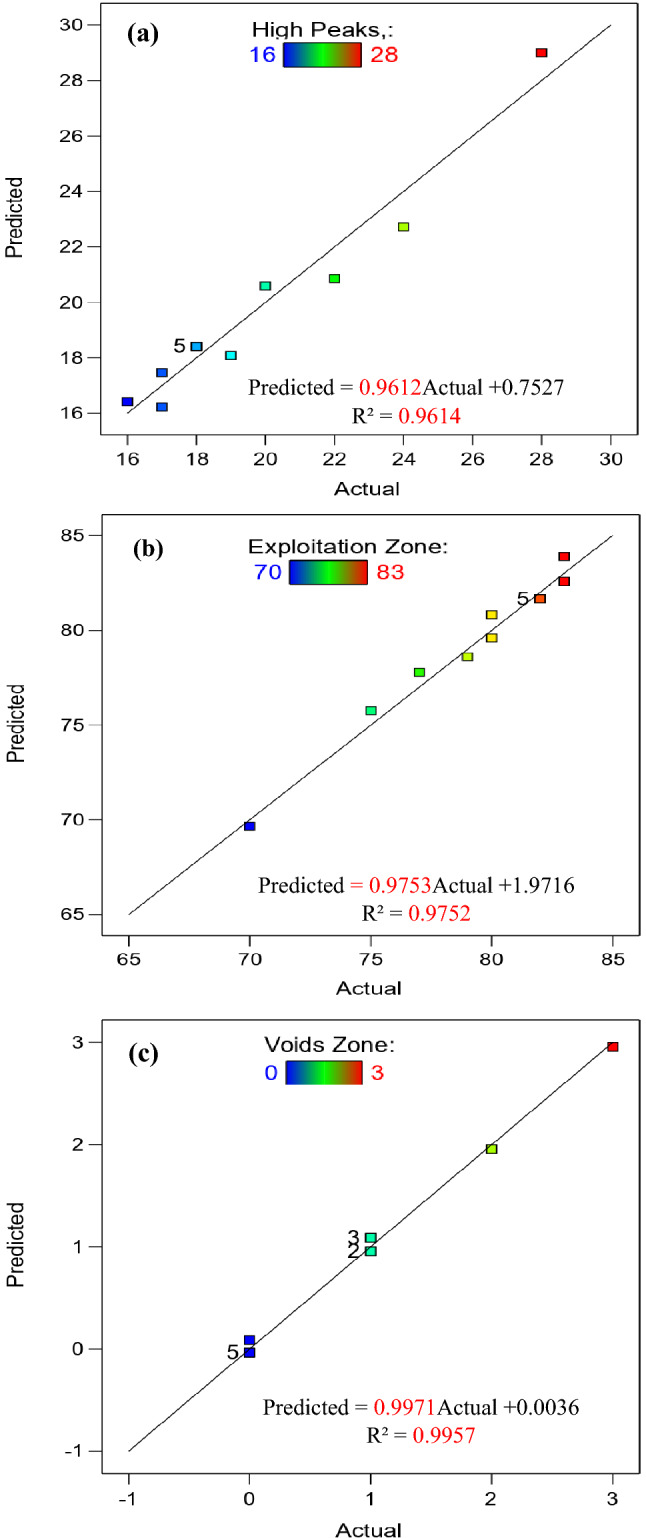
Relationship between actual and predicted Abbott Firestone zones of hot-rolled.

**Figure 17:**
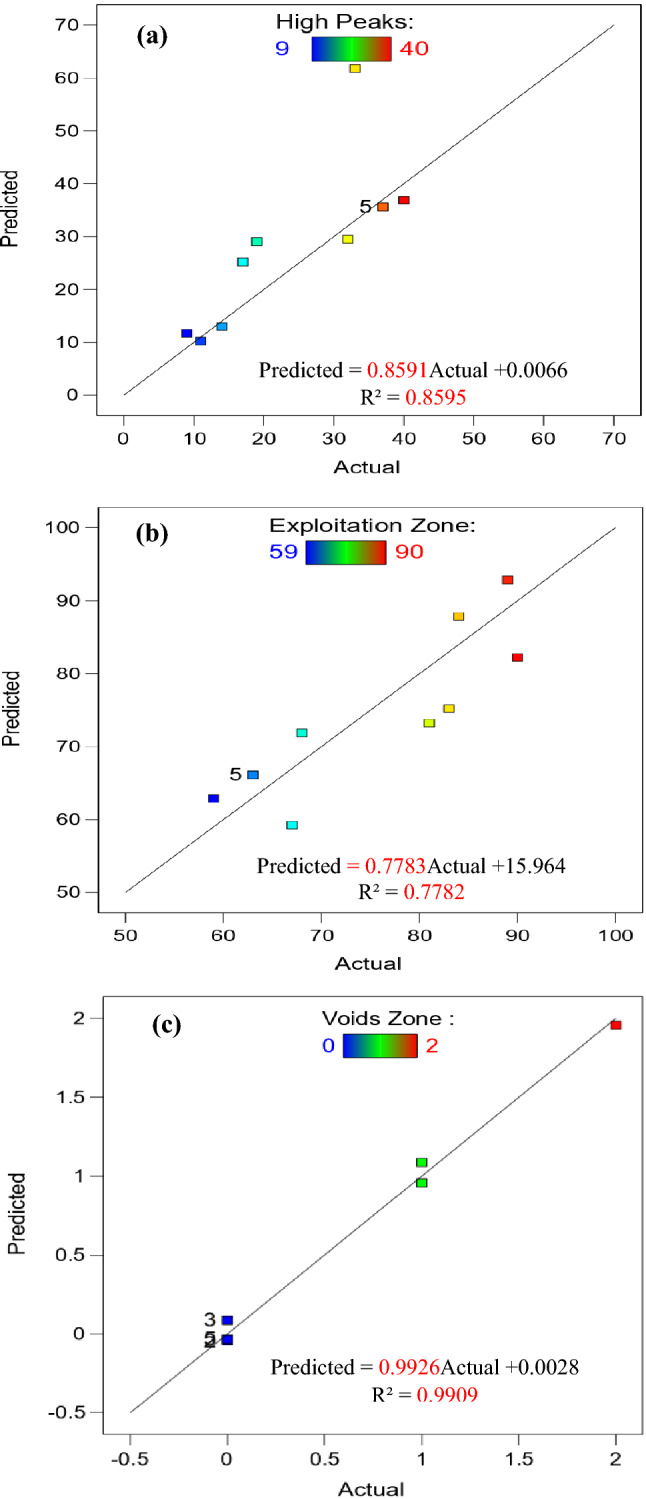
Relationship between actual and predicted Abbott Firestone zones of QAM_f_.

Figure 14 shows 3D surface plot of Abbott Firestone zones (high peaks, exploitation, and voids) of QAM_f_ steel. Considering the effect of velocity (V) at two different values of pressure (P), which were at 0.5 and 2.5 MPa, it can be observed that P effect was stronger in the second case on the high peaks zone, Fig. 14a. In the contrary for exploitation and voids zones, it can be observed that P effect was stronger in the first case, Fig. 14b,c. Figure 15 shows a contour map of QAM_f_ steel. At increasing velocity and pressure gradually increases high peaks (Fig. 15a), while at decreasing velocity and pressure exhibits an increase voids zone (Fig. 15c). However, at low and high velocities, decreasing pressure gradually increases the exploitation zone (Fig. 15b). Figures 16 and 17 show relationship between actual and predicted Abbott Firestone zones (high peaks, exploitation, and voids).

## Conclusions

In this study, the influences of applied pressure and running velocity (input factors) on wear rate as well as Abbott Firestone zones after hot-rolled and QAM_f_ of low carbon steel (0.16C) were investigated in an experimental setting with the DOE-based CCD technique. Based on the results of the current experiments and modeling, the following are the conclusions:

1. Wear rate increases with increased pressure and velocity. Pressure had the biggest effect on the wear rate behavior of the two variables investigated. On the other hand, Abbott Firestone zones were built by EDT.
2. QAM_f_ process relatively decreased wear rate of 0.16C steel compared to hot rolling process.
3. The best wear rate models and Abbott Firestone zones offered precise data that could be approximated, saving time and cost.
4. RSM model was used to find the best wear parameter values for achieving the lowest wear rate.
5. Predictive wear model using RSM can be applicable for a certain wear system to estimate its wear rate.
6. Predicted results coincide well with the experimental findings, indicating that the developed models can be used to accurately forecast wear behavior and Abbott Firestone zones.

## Data Availability

All data generated or analyzed during this study are included in this published article.
